# Trends in unicompartmental knee arthroplasty among 138 international experienced arthroplasty knee surgeons

**DOI:** 10.1016/j.heliyon.2024.e24307

**Published:** 2024-01-12

**Authors:** Matteo Marullo, Reha N. Tandogan, Nanne Kort, Amit Meena, Manish Attri, Bruce Gomberg, Riccardo D'Ambrosi, EKA Small Implants Focus Group

**Affiliations:** a IRCCS Ospedale Galeazzi – Sant’Ambrogio, Milan, Italy; b Department of Orthopaedics and Traumatology, Emsey Hospital, Istanbul, Turkey; c Department of Orthopaedics and Traumatology, Halic University Istanbul & Cankaya Orthopedics, Ankara, Turkey; d CortoClinics, Nederweert, Netherlands; e Gelenkpunkt – Sports and Joint Surgery, FIFA Medical Centre of Excellence, Innsbruck, Austria; f Central Institute of Orthopaedics, Vardhman Mahavir Medical College and Safdarjung Hospital, New Delhi, India; g Northern Light Mercy Orthopaedics, Portland, ME, USA; h Università Degli Studi di Milano, Dipartimento di Scienze Biomediche per La Salute, Milan, Italy

**Keywords:** Unicompartmental knee replacement, UKA, Knee osteoarthritis, Indications, Survey

## Abstract

**Purpose:**

Unicompartmental knee arthroplasty (UKA) is an established option for treating isolated unicompartmental knee osteoarthritis (OA), but controversies still exist about patient selection, indications, perioperative management and alignment goals. This survey was designed to understand the current trends of experienced arthroplasty knee surgeons performing UKA.

**Methods:**

An online questionnaire was created with SurveyMonkey**®** to assess global tendencies in the utilization of UKA. A link to the survey was shared with all ESSKA (European Society for Sports Traumatology, Knee Surgery and Arthroscopy) members. The questionnaire consisted of free and multiple-choice questions and was divided into four sections: demographic information, the surgical activity of the respondents, indications for surgery and postoperative alignment goals.

**Results:**

A total of 138 ESSKA members from 34 different countries completed the survey. A total of 81 % of the responders performed fewer than 50 UKAs per year; 53 % of UKAs represented less than 20 % of their knee replacements; 71 % used mainly or only fixed-bearing implants; 81 % performed UKA in a shorter time compared to TKA; and 61 % and 72 % were interested in custom-made UKA and robotics, respectively. Thirty-six percent considered a minimum postoperative alignment of 0° for medial UKA, and 32 % considered 10° as the maximum valgus deformity for lateral UKA. Fifty-five percent had no minimum age cut-off, 47 % had no BMI cut-off, and 57 % believed TKA was better than UKA in knees with concomitant high-grade patellofemoral OA. Approximately 50 % of the surgeons desired a coronal alignment that was the same as the predegeneration alignment.

**Conclusion:**

A high level of agreement was reached regarding the following: preference for fixed-bearing UKAs, lower surgical time for UKA compared to TKA, interest in custom-made and robotic UKAs, no age and weight cut-off, TKA preferred in the presence of patellofemoral OA, and a final alignment goal of the predegenerative state both for medial and lateral. There was no agreement regarding length of stay, rehabilitation protocol, preoperative varus and valgus cut-off values, and treatment in cases of absence of anterior cruciate ligament or previous osteotomy.

**Level of Evidence:** Level IV.

## 1 Introduction

Unicompartmental knee arthroplasty (UKA) has been proven to be an effective treatment for isolated compartmental knee osteoarthritis (OA) in selected patients [[1], [2], [3]]. UKA presents several advantages over total knee arthroplasty (TKA), such as faster recovery, fewer complications and better function. However, higher rates of failure compared to TKA have been reported in national registries for UKA [[4], [5], [6]]. For this reason, many surgeons are still reluctant to perform this procedure. On some account, 85 % of knees with OA have isolated medial compartment involvement, but only between 5 % and 10 % of the surgical interventions are UKA [7].

The characteristics that most affect survivorship are proper patient selection and ideal pre- and postoperative coronal alignment [8]. In the early adoption phase of UKA, Kozinn and Scott proposed strict criteria for patient selection to optimize implant survivorship. These included patient age less than 60 years, weight less than 82 kg, angular deformity less than 15° (passively correctable to the neutral), flexion contracture less than 5°, and range of motion more than 90° [9]. Some of these criteria have been updated, and there is a growing interest in new technologies such as digital aids and robotics to improve outcomes [[10], [11], [12], [13]]. Despite a large volume of studies in the literature on UKA, controversies about patient selection criteria remain unanswered. This survey was designed to investigate the current trends in UKA usage with a particular focus on indications, pre- and postoperative alignment, and interest in new technologies utilizing responses from experienced unicompartmental knee surgeons. According to literature, were considered experienced unicompartmental knee surgeons who performed at least >20 % UKA (compared to TKA) or 12 UKA procedures/year [14].

## 2 Material and methods

Ethical approval was obtained by Ethical Committe. of IRCCS San Raffaele Hospital, Milan, Italy – 236/2017). An online questionnaire (https://it.surveymonkey.com/stories/SM-7TLFZ3S2/) was created with SurveyMonkey® (Momentive Inc. San Mateo, California, United States of America) to assess global tendencies in the usage of UKA [15]. A link to the survey was shared with all ESSKA (European Society for Sports Traumatology, Knee Surgery and Arthroscopy) members by mail. Participation in the study was voluntary and with no remuneration. No e-mail addresses or other identifying data were collected from the participants. The participants were able to respond only once from the same device.

### 2.1 Questionnaire structure

The questionnaire consisted of free and multiple-choice questions and was divided into four sections. The first section had three questions regarding the participants’ demographic information (age, gender and nationality). The second section consisted of 13 questions regarding their surgical activity. The third section contained 8 questions concerning the indications for replacement surgery, and the fourth section included 2 questions about postoperative alignment. A complete version of the survey and answers can be found in Appendix A, Appendix A.

## 3 Results

All percentages were rounded to the nearest whole number for ease of reading.

### 3.1 Demographics of the respondents

The survey was sent to all ESSKA members, and 138 completed the survey. The age range for the majority of the participants was between 35 and 44 years (36 %). The surgeons were mostly males (97 %), and the majority of the respondents were Italian surgeons (19 %), followed by German (9 %) and Spanish (9 %) surgeons; however, surgeons from 34 different countries completed the survey. The surgeons’ years of expertise in knee replacement ranged from 2 years to more than 50 years (17 ± 11 years).

**How much of your activity does knee replacement surgery represent?**

Forty-two percent of the participants had more than 50 % of their surgical activity as knee replacement surgery, 26 % between 31 and 50 %, 26 % between 11 and 30 % and only 6 % under 10 %.

**How many partial knee replacements (UKA, PFA, BiUKA, BCA) do you perform each year?**

Thirty-three percent of the participants performed between 11 and 30 UKAs per year, 22 % performed 31–50 UKAs per year, 26 % performed fewer than 10, and 19 % performed more than 50.

**What is your ratio of partial knee replacements compared to all your knee replacements?**

Five percent of the surgeons did not perform UKA at all. Twenty-eight percent performed less than 10 %; 25 % performed up to 20 % UKAs; 16 % performed up to 30 % UKAs; 14 % performed up to 40 % UKAs; 5 % surgeons performed up to 50 % UKAs; and only 8 % surgeons performed more than 50 % UKAs in comparison to their total knee replacement population.

**What is your ratio of medial UKA according to all your partial knee replacements?**

Twenty-one percent of the surgeons exclusively performed medial UKAs, 33 % performed more than 91 % medial UKAs, 21 % performed more than 81 % medial UKAs, and 15 % performed more than 61 %, whereas only 11 % of the surgeons performed less than 60 % medial UKAs as a proportion of partial replacements.

**Do you utilize fixed-bearing UKA or mobile-bearing UKA?**

Fifty-six percent of the surgeons utilized only fixed-bearing UKA implants; 15 % used mainly fixed-bearing UKA implants; 7 % responded that they equally preferred both types of implants; 10 % mainly used mobile-bearing UKA implants, whereas 12 % of surgeons used only mobile-bearing implants.

**What is the rationale behind choosing the type of implant for UKA?**

Twenty-seven percent believed that a certain type of implant had better clinical outcomes; 35 % surgeons used a specific implant type because they were trained on its use; 12 % believed that their chosen UKA implant had lower failure rates; 9 % believed their preferred implant type had better kinematics, whereas 11 % of the surgeons had other reasons for choosing the implants, such as easier surgical technique, availability of a robotic aid, availability of the implants, reproducibility, and reduced surgical time with the fixed-bearing implants. Some choose implants on the basis of the site of OA (e.g., mobile-bearing for medial compartment OA and fixed-bearing for lateral compartment OA).

**Is your surgical time for a UKA shorter or longer compared to the one for a TKA?**

Sixteen percent of surgeons claimed that their surgical time was reduced by more than 30 min when performing UKA; 65 % believed that the surgical time for UKA was shorter than that for TKA by at least 5–30 min; 13 % of surgeons said that the surgical time was the same for both procedures; 3 % of surgeons took longer (5–30 min) to perform UKA, whereas 3 % of surgeons took much longer (>30 min) to perform UKA than TKA.

**How long does a patient stay in the hospital after their UKA surgery?**

Six percent of surgeons responded that they performed UKA as an outpatient procedure. Thirty percent discharged their patients after one night's stay in the hospital; 30 % kept their patients admitted for two nights post-UKA surgery; 23 % discharged their patients 3 nights after the surgery; 9 % discharged their patients after 4–6 days of hospital stay, and 2 % surgeons kept the patients hospitalized for more than six days after the UKA surgery.

**Do you use the same rehabilitation protocol for UKA as for TKA?**

Forty-four percent of surgeons used the same protocol for both UKA and TKA. Fifty-five percent of surgeons used a shorter rehabilitation protocol for UKA than for TKA, whereas 1 % used a longer rehabilitation protocol for UKA than for TKA.

**What is you included in your perioperative pain management protocol (more than one answer is possible)?**

Forty percent of surgeons used preoperative oral medications, 43 % used preoperative regional blocks, 75 % utilized periarticular injections or local infiltration analgesia, and 20 % surgeons made use of continuous postoperative regional blocks for pain management in patients undergoing UKA.

**Are you interested in custom-made UKA?**

Five percent of surgeons claimed that they had used custom-made UKA; 57 % stated that they were interested in using custom-made UKA; 26 % did not believe that custom-made UKA would be useful; 5 % reported that they had tried it but did not believe it could help them; and 9 % of the surgeons stated that they were not interested in custom-made UKA.

**Are you interested in robotics applied to UKA?**

Fifteen percent of surgeons reported that they used robotics for UKA surgery; 57 % were interested in using robotics in UKA; 18 % believed that robotics was not useful for UKA surgery; 4 % reported that they had tried it but did not find it useful; and 7 % were not interested in robotics applied to UKA.

**What is your minimum varus alignment for medial UKA?**

Nine percent of surgeons did not perform a whole leg standing X-ray before UKA; 10 % were not worried about the preoperative alignment; 36 % considered a minimum preoperative alignment of zero degrees with no valgus before UKA; 31 % considered a minimum varus alignment for medial UKA as 3°; and 14 % considered a minimum varus alignment for medial UKA as 5°.

**Which is your varus deformity cut-off for considering a medial UKA?**

Eighteen percent of surgeons said that they regarded 5° of varus deformity as the cut-off; 28 % took this value as 10°; 30 % considered 15° of varus deformity that was reducible as the cut-off value; 8 % said that for them, the limit of varus deformity was 15°, and for 16 % surgeons, there was no cut-off value when considering medial UKA.

**Which is your valgus deformity cut-off for considering a lateral UKA?**

26 % of surgeons said that they regarded 5° of valgus deformity as the cut-off for considering a lateral UKA; 32 % took this value as 10°; 18 % considered a reducible 15° valgus deformity as the cut-off value; 8 % noted that for them the limit of the valgus deformity was 15° and for 17 % surgeons, there was no cut-off value while considering a lateral UKA.

**Do you have a minimum age limit cut-off for considering a small implant in end-stage compartmental OA?**

Fifty-five percent of surgeons had no minimum age limit cut-off; 8 % had age limit cut-offs only for patellofemoral replacements; 3 % of surgeons had an age limit cut-off of 30 years; 14 % had a cut-off of 40 years; 18 % considered a small implant in patients aged more than 50 years, whereas 3 % had an age of >60 years for considering a UKA in end-stage single compartment OA.

**Do you have a strict cut-off on preoperative BMI for implanting a small implant?**

Forty-seven percent of the respondents had no BMI cut-off for implanting a UKA; 30 % of surgeons were strict about not using small implants for patients with BMI>35 kg/m^2^; 19 % had a BMI cut-off value of >30 kg/m^2,^ and 4 % did not use small implants in patients with BMI >25 kg/m^2^.

**Do you believe it is feasible to implant a UKA in an ACL-deficient knee?**

Seventeen percent of surgeons did not believe that it was feasible to implant a UKA in an ACL-deficient knee; 4 % considered it to be always feasible to implant a UKA in an ACL-deficient knee; 22 % of surgeons advised implanting a UKA in an ACL-deficient knee only in the absence of subjective instability; 37 % surgeons considered the implantation of a UKA in an ACL-deficient knee only in older patients with primary anteromedial OA and secondary degenerative ACL deficiency, while only 20 % of the surgeons agreed to implant UKA in an ACL-deficient knee only if associated with an ACL reconstruction.

**Do you consider previous high tibial osteotomy a contraindication for medial UKA?**

Twenty-five percent of surgeons considered previous high tibial osteotomy a contraindication for medial UKA; 38 % of surgeons did not, and 37 % believed that UKA was feasible after HTO only if HTO undercorrected the coronal deformity, allowing postoperative alignment after medial UKA to remain in varus.

**Do you believe it is feasible to implant an isolated UKA in knee with a concomitant high grade patellofemoral OA?**

Fifty-seven percent of the respondents believed that TKA was a better solution than isolated UKA in a knee with concomitant high-grade patellofemoral OA; 17 % believed that in such cases, UKA with PFA was a better option; 19 % responded that patellofemoral OA was not a contraindication to UKA; 3 % thought that isolated UKA could be performed in a knee with concomitant high-grade patellofemoral OA only if mobile-bearing UKA was utilized, whereas 4 % thought that UKA was feasible in a knee with concomitant high-grade patellofemoral OA in male patients.

**Which is your desired coronal alignment after a medial UKA (evaluated with long standing X-rays)?**

Four percent of the surgeons desired a coronal alignment that was the same as the preoperative alignment; 51 % desired a coronal alignment that was the same as the predegeneration alignment to preserve the constitutional varus; 15 % surgeons desired a neutral coronal plane alignment; 28 % desired 1–5° varus; 1 % thought 5–10° varus was desirable and 1 % surgeons desired a valgus alignment of 0–5°.

**Which is your desired coronal alignment after lateral UKA (evaluated with long-standing X-rays)?**

Six percent of surgeons desired a coronal alignment that was the same as the preoperative alignment; 49 % desired a coronal alignment that was the same as the predegeneration alignment to preserve the constitutional valgus; 23 % desired a neutral coronal plane alignment; 22 % surgeons desired 1–5° valgus; and 1 % of surgeons thought 5–10° valgus was desirable.

## 4 Discussion

The most important finding of the present study was that at the there is still significant skepticism and low confidence levels in the use of UKA, even though improvements in surgical techniques and implant design have resulted in improved UKA outcomes [1,7,16]. Despite being a less invasive procedure and having multiple advantages over TKA, the usage of UKA remains relatively low. Approximately 47 % of patients with knee OA have been reported to be candidates for UKA [1,7,16]. However, utilization of UKA is only 5–8% [[17], [18], [19]]. In our survey, 53 % of respondents utilized UKA for less than 20 % of their knee replacements. This skepticism regarding UKA is likely due to two factors. First, there is a high revision rate of UKA reported in the national joint registries (NJRs) [20]. The revision rate for UKA is 3.2 times higher than that for TKA in NJR for Wales, England and Northern Ireland [21]. The high revision rates of UKA reported in national registries conflict with those reported by cohort studies performed by experienced surgeons in high-volume centres, which report comparable survivorship between UKA and TKA [22]. This difference probably reflects the complexity of the right indication and the proper surgical technique for UKA. Second, the UKA procedure has a steeper learning curve than TKA [23].

Another important aspect is the caseload phenomenon with UKA; in fact, Mohammad et al. demonstrated that caseload had a profound effect on implant survival. Low-volume surgeons had a high revision rate with cemented or cementless fixation and therefore should consider either stopping or doing more UKA procedures. High-volume surgeons performing cementless UKA demonstrated a 10-year survival rate of 97.5 %, which was similar to that reported in registries for the best-performing TKAs [6].

Likewise, Hamilton et al. reported that to achieve optimum results, surgeons, whether high or low caseload, should adhere to the recommended indications such that ≥20 %, or ideally >30 % of their knee arthroplasties are UKA. If they do this, then they can expect to achieve results similar to those of the long-term series, which all had high usage (>20 %) and an average 10-year survival of 94 % [24].

Finally, Liddle et al. confirmed he importance of surgical caseload in determining the survival of UKR and, to a lesser extent, TKR. The reasons for this effect are complex and not fully explained by variables recorded in the National Joint Registry; however, the patient selection and revision threshold of lower-volume surgeons may be a factor [25].

Regarding the ratio between medial and lateral or isolated patellofemoral, our results are in line with the current literature [26,27]. Lateral UKA and patellofemoral arthroplasty are less commonly performed than medial UKA, mainly due to their lower incidence and complex surgical techniques [[26], [27], [28]].

The majority of the surgeons in our survey were comfortable with fixed-bearing implants (56 %). Recent meta-analyses showed no differences in clinical patient-reported outcomes or overall revision rates between mobile-bearing and fixed-bearing implants [18,29]. There are several possible explanations for the higher preference for fixed-bearing UKAs in this survey: fixed-bearing UKAs are easier to learn (shortening the learning curve), are reproducible in nondeveloper hands and are easier to balance [18,29]. They can also be more bone-preserving and easier to revise. Last, some robotic systems are specific for fixed-bearing UKAs, while there is no robotic system available to pair with mobile bearing systems.

Almost all the surgeons in our survey confirmed that performing a UKA takes less surgical time than performing a TKA, which is in agreement with the findings of Migliorini et al. [30].

Regarding the length of stay after UKA, there was no agreement: in fact, 30 % of the surgeons performed outpatient surgery, while 23 % reported a recovery of 3 nights. The same day discharge after UKA has been found to be safe and successful when done in an outpatient facility [[31], [32], [33]]. A few studies assessing same-day discharge from an outpatient surgical setting in a nonselected cohort have reported 100 % success with low readmission rates and postoperative complications [[32], [33], [34]]. In our cohort, differences in length of stay are explained by differences in healthcare organization among countries and regions. Same-day discharge may require home care by physical therapists, nurses, or other qualified medical personnel within 24 h, something simply not feasible in all countries. In other countries, there is a financial disincentive for same-day discharge. In a nonselected cohort, same-day discharge was only achievable for 6.4 %–9.7 % of patients, which is likely a more representative cohort of patients in the national registry [35,36].

There was no agreement about rehabilitation protocols. UKA has several advantages over TKA, including preservation of more native tissue, a smaller incision, decreased blood loss, less perioperative morbidity, superior proprioception, reduced pain, shorter hospitalization, greater range of motion (ROM), and a more rapid and shorter rehabilitation course [37,38]. However, our survey found highly variable practices of postoperative care, with 44 % of surgeons employing the same protocols as for TKA.

Seventy-four percent of the respondents routinely used periarticular injections or local infiltration analgesia in UKA. This is in line with the most recent publications about UKA performed in an outpatient and enhanced recovery setting [35,39].

There is great interest in custom-made UKA implants and robotics applied to UKA. Recent studies have shown that robotic surgery improves component positioning and the accuracy of coronal alignment. Considering that malpositioning and under- or overcorrection are the main causes of UKA failure, robotic assistance could potentially be very helpful in UKA. However, to date, robotic surgery has not resulted in improved clinical scores, patient satisfaction or implant survivorship [40,41].

Approximately 58 % of the respondents had a cut-off of less than 10° of valgus deformity for considering lateral UKA, and approximately 45 % had a cut-off of less than 10° of varus deformity for a medial UKA. These findings are interesting because these limits are even more restrictive than the classical Kozinn and Scott indications [9]. Only 16 % of the respondents had no cut-off limits for medial or lateral UKA.

Approximately 50 % of the respondents had no BMI cut-off for implanting a UKA. A recent meta-analysis showed that obesity did not influence clinical and most functional outcomes after UKA, even if UKA in obese and severely obese patients had a lower 10-year survivorship than that in nonobese patients [42]. In total, lower survivorship in obese patients compared to nonobese patients was seen even for TKA, and obesity seems to have a lower impact on the revision rate after UKA than after TKA [43].

Most of the respondents did not consider younger age as a contraindication for UKA. Improvements in UKA survivorship and clinical outcomes, even in very young patients, could explain the disagreement with Kozinn and Scott's criteria [44,45].

Almost 40 % of the respondents confirmed that a previous HTO is not a contraindication for UKA. Performing knee arthroplasty following a previous HTO is a demanding procedure due to considerable soft tissue scarring, a potential decrease in bone stock, and altered tibial slope and height of the patella [46,47]. Patients with prior HTO develop OA at an earlier stage in life, and the knee becomes more susceptible to increased complications and inferior outcomes [46,47].

A high level of agreement was found in our sample for the preference for TKA in the case of associated patellofemoral OA. The literature suggests that OA on the medial facet of the patella should not be considered a contraindication for UKA since UKA unloads this area and does not lead to worse outcomes or survivorship at 10–15 years. In contrast, full-thickness lateral patellofemoral cartilage loss should be considered a contraindication for UKA [48].

For the final alignment, approximately 50 % of the respondents aimed for a coronal alignment similar to the predegenerative state for both medial and lateral UKA. In medial UKA, slight postoperative varus of 1–4° showed the best functional results and survivorship [49,50].

Slaven et al. demonstrated that patients with well-functioning UKAs at 10 years exhibited mild varus mechanical alignment of approximately 4°, whereas patients revised for progression of osteoarthritis averaged more valgus and those revised for loosening or subsidence averaged more varus. The optimal mechanical alignment for medial fixed-bearing UKA survival with contemporary polyethylene is likely slight varus [51].

Similarly, Kennedy et al. investigated the relationship between postoperative limb alignment and postoperative patient-reported outcome and implant revision rate. Marked postoperative varus mechanical alignment of an estimated 10° was present in 8 %, and mild varus of about 5° was present in 35 %. Increasing varus alignment was associated with an increasing percentage of good or excellent clinical outcomes, but otherwise there were no significant differences between alignment groups in patient-reported outcome or revision rate [52].

Finally, Gulati et al. assessed the effect of component malalignment on outcome of Oxford Unicompartmental Knee Replacement concluding that because of the spherical femoral component, the Oxford UKR is tolerant to femoral mal-alignment of 10° and tibial mal-alignment of 5° [53].

However, in lateral UKA, mild valgus alignment (less than 4°) has been linked to lower clinical and functional scores and a higher revision rate than moderate valgus alignment (greater than 4°).

Consequently, greater undercorrection appears to be necessary for lateral UKA compared to medial UKA [54].

This survey showed that the routine practice of the respondents, who are mainly specialized knee surgeons, was not in line with the classical Kozinn and Scott criteria in performing unicompartmental knee arthroplasty. If Kozinn and Scott criteria were strictly applied, only 6–12 % of patients with knee osteoarthritis would be eligible for UKA; however, when the indications are expanded, the candidacy for UKA could increase up to 50 % [7]. As stated by Murray et al. this could not only increase patients’ functional results but also lower the complication rate, decrease the cost, and decrease mortality after the procedure [17].

This study has several limitations. First, the population that chose to complete the survey represents a small subset of the orthopaedic surgeons involved in knee pathology; therefore, the results of the present survey are not generalizable. Conversely, performing the survey on highly specialized surgeons suggests which aspects of patient selection and indications for UKA may need to be further reviewed. Second, given the intrinsic nature of an online survey, the respondents were limited in their choice of responses and had no opportunity to discuss their responses in detail. Moreover, the questionnaire did not distinguish the different aetiologies of OA (idiopathic, postmeniscectomy, traumatic). Finally, the questions of the present study have been developed based on expert opinion rather than through a validated methodology.

## 5 Conclusions

A high level of agreement was reached regarding the following: preference for fixed-bearing UKAs, lower surgical time for UKA compared to TKA, interest in custom-made and robotic UKAs, no age and weight cut-off, TKA preferred in the presence of patellofemoral OA, and a final alignment goal of the predegenerative state both for medial and lateral. There was no agreement regarding length of stay, rehabilitation protocol, preoperative varus and valgus cut-off values, and treatment in cases of absence of anterior cruciate ligament or previous osteotomy.

## Abbreviations

Not Applicable.

## Ethical approval

Ethical approval was obtained by Ethical Committe. of IRCCS San Raffaele Hospital, Milan, Italy – 236/2017.

## Consent to participate

Participation to the study was voluntary and with no remuneration. No e-mail addresses or other identifying data were collected from all participants.

## Consent to publish

All authors consent to the publication of the manuscript.

## Availability of supporting data

Raw data have been submitted in the form of an appendix (Appenidx 2) and are available upon reasonable request to the corresponding author.

## Funding

None.

## CRediT authorship contribution statement

**Matteo Marullo:** Writing – review & editing, Investigation, Data curation, Conceptualization. **Reha N. Tandogan:** Writing – review & editing, Supervision, Conceptualization. **Nanne Kort:** Writing – review & editing, Supervision. **Amit Meena:** Writing – review & editing, Writing – original draft, Methodology. **Manish Attri:** Writing – review & editing, Writing – original draft. **Bruce Gomberg:** Writing – review & editing, Supervision, Conceptualization. **Riccardo D'Ambrosi:** Writing – review & editing, Writing – original draft, Supervision, Methodology, Data curation, Conceptualization.

## Declaration of competing interest

The authors declare that they have no known competing financial interests or personal relationships that could have appeared to influence the work reported in this paper..

## Footnotes

Appendix A Supplementary data to this article can be found online at https://doi.org/10.1016/j.heliyon.2024.e24307.
