# Supplementary material for: Trends in unicompartmental knee arthroplasty among 138 international experienced arthroplasty knee surgeons
Source: Heliyon. 2024 Jan 12;10(2):e24307. doi: 10.1016/j.heliyon.2024.e24307 (PMC10830546; doi:10.1016/j.heliyon.2024.e24307)
Supplement: Multimedia component 1 [file mmc1.docx]

Questionnaire

1. How old are you?

- Under 18
- 18-24
- 25-34
- 35-44
- 45-54
- 55-64
- 65+

1. Gender?

- Male
- Female

1. What is your nationality?

- Open answer

1. How many years of expertise in knee replacement do you have?

- Open answer

1. How much of your activity does knee replacement surgery represent?
   - <10%
   - 11-30%
   - 31-50%
   - 51-70%
   - 71-90%
   - >91%
2. How many partial knee replacements (UKA, PFA, BiUKA, BCA) do you perform each year?
   - 0
   - <10
   - 11-30
   - 31-50
   - 51-100
   - >100
3. What is your ratio of partial knee replacements compared to all your knee replacements?
   - 0%
   - 1-10%
   - 11-20%
   - 21-30%
   - 31-40%
   - 41-50%
   - >51%
4. What is your ratio of medial UKA according to all your PARTIAL knee replacements?

- 100%
- 91-100%
- 81-90%
- 61-80%
- <60%

1. Do you utilize fixed-bearing UKA or mobile-bearing UKA?

- Only fixed-bearing
- Mainly fixed-bearing
- 50%-50%
- Mainly mobile-bearing
- Only mobile-bearing

1. Why?

- Better clinical outcome
- I was trained on it
- Lower failure rate
- Higher risk of dislocation
- Better kinematics
- Other (please specify)

1. Is your surgical time for a UKA shorter or longer compared to the one for a TKA?

- Much shorted (>30 minutes less)
- Shorted (5-30 minutes less)
- Same
- Longer (5-30 minutes difference)
- Much longer (>30 minutes difference)

1. How long does your patient stay in the hospital after unicompartmental knee replacement surgery?

- No nights – Outpatient
- One night
- Two nights
- Three nights
- Four to six nights
- More than six nights

1. Do you use the same rehabilitation protocol for a UKA as for a TKA?
   - Yes
   - No -shorter rehabilitation for UKA
   - No – longer rehabilitation for UKA
2. What do you include in your perioperative pain management protocol (more than one answer is possible)
   - Pre-op oral medicine
   - Pre-op regional blocks
   - Periarticular injections or local infiltration analgesia
   - Continuous post-op regional block
3. Are you interested in custom-made UKA?
   - Yes, I use it
   - Yes, I could use it
   - No, I don’t believe it would be useful
   - I have tried it, but I did not believe it would help me
   - Not at all
4. Are you interested in robotics applied to UKA?
   - Yes, I use it
   - Yes, I could use it
   - No, I don’t believe it would be useful
   - I have tried it, but I did not believe it would help me
   - Not at all
5. What is your minimum varus alignment for medial UKA?
   - I do not perform whole leg standing x-ray before UKA
   - I don´t care about preoperative alignment
   - Minimum 0°, no valgus
   - Minimum 3° varus
   - Minimum 5° varus
6. Which is your varus deformity cut-off for considering a medial UKA?
   - 5°
   - 10°
   - 15° reducible
   - 15°
   - No cut off
7. Which is your valgus deformity cut-off for considering a lateral UKA?

- 5°
- 10°
- 15° reducible
- 15°
- No cut off

1. Do you have a minimum age cut-off for considering a small implant in end stage compartimental OA?

- No
- No cut-off, only for patellofemoral replacements
- Yes, over 30 years old
- Yes, over 40 years old
- Yes, over 50 years old
- Yes, over 60 years old

1. Do you have a hard cut-off on preoperative Body Mass Index (BMI) for implanting a small implant?

- No
- Yes, no small implants if BMI > 35 kg/m^2
- Yes, no small implants if BMI > 30 kg/m^2
- Yes, no small implants if BMI > 25 kg/m^2

1. Do you believe it is feasible to implant a UKA in an ACL-deficient knee?

- No, never
- Yes, always
- Only in absence of subjective instability
- Only in older patients with primary antero(medial) OA and secondary degenerative ACL deficiency
- Only if associated with an ACL reconstruction

1. Do you believe it is feasible to implant an isolated UKA in a knee with a concomitant high-grade patellofemoral OA?

- No, better a TKA
- No, better a UKA+PFA
- Yes. no matter about patellofemoral OA
- Yes, if a mobile-bearing UKA is utilized
- Yes, only in male
- Yes, only in female

1. Which is your desired coronal alignment after a medial UKA (evaluated with long-standing X-rays)?

- The same as preoperative
- The same as pre-degeneration (therefore preserving constitutional varus)
- Neutral (0°)
- 1-5° varus
- 5-10° varus
- 1-5° valgus

1. Which is your desired coronal alignment after a lateral UKA (evaluated with long-standing X-rays)?

- The same as preoperative
- The same as pre-degeneration (therefore preserving constitutional valgus)
- Neutral (0°)
- 1-5° valgus
- 5-10° valgus
- 1-5° varus
