# Supplementary material for: Trends in unicompartmental knee arthroplasty among 138 international experienced arthroplasty knee surgeons
Source: Heliyon. 2024 Jan 12;10(2):e24307. doi: 10.1016/j.heliyon.2024.e24307 (PMC10830546; doi:10.1016/j.heliyon.2024.e24307)
Supplement: Multimedia component 2 [file mmc2.docx]

| 1. **How old are you?** | |  |
| --- | --- | --- |
| Answer Choices | Responses | |
| Under 18 | 0,00% | 0 |
| 18-24 | 1,55% | 2 |
| 25-34 | 9,30% | 12 |
| 35-44 | 35,66% | 46 |
| 45-54 | 23,26% | 30 |
| 55-64 | 20,93% | 27 |
| 65+ | 9,30% | 12 |
|  | **Answered** | **129** |
|  | **Skipped** | **1** |

| 1. **Gender?** |  |  |
| --- | --- | --- |
| Answer Choices | Responses | |
| Male | 96,90% | 125 |
| Female | 3,10% | 4 |
|  | **Answered** | **129** |
|  | **Skipped** | **1** |

| 1. **What is your nationality?** |  |  |
| --- | --- | --- |
| Answer Choices | Responses | |
| American | 2,33% | 3 |
| Austrian | 0,78% | 1 |
| Belgian | 7,75% | 10 |
| British | 4,65% | 6 |
| Chilean | 2,33% | 3 |
| Croatian | 0,78% | 1 |
| Cuban | 0,78% | 1 |
| Danish | 1,55% | 2 |
| Dutch | 3,88% | 5 |
| English | 0,78% | 1 |
| French | 3,88% | 5 |
| German | 9,30% | 12 |
| Greek | 7,75% | 10 |
| Hungarian | 0,78% | 1 |
| Italian | 18,60% | 24 |
| Mexican | 0,78% | 1 |
| New Zealander | 0,78% | 1 |
| Pakistani | 0,78% | 1 |
| Peruvian | 0,78% | 1 |
| Polish | 1,55% | 2 |
| Portuguese | 3,10% | 4 |
| Romanian | 3,10% | 4 |
| Russian | 2,33% | 3 |
| Singaporean | 0,78% | 1 |
| Slovak | 0,78% | 1 |
| Slovenian | 0,78% | 1 |
| Somali | 0,00% | 0 |
| South African | 0,78% | 1 |
| South Korean | 0,78% | 1 |
| Spanish | 8,53% | 11 |
| Swedish | 1,55% | 2 |
| Swiss | 2,33% | 3 |
| Turkish | 2,33% | 3 |
| Ukrainian | 2,33% | 3 |
|  | **Answered** | **129** |
|  | **Skipped** | **1** |

| 1. **How many years of expertise in knee replacement do you have?** | |
| --- | --- |
| **Answered 106** |  |
| **Skipped** |  |
| 24 |  |
|  |  |
| Respondents | Responses |
| 1 | 10 |
| 2 | 10 |
| 3 | 27 |
| 4 | 35 |
| 5 | 14 |
| 6 | 17 |
| 7 | 5 |
| 8 | 22 |
| 9 | More than 25 years |
| 10 | 8 |
| 11 | 25 |
| 12 | 7 |
| 13 | 5 |
| 14 | 30 |
| 15 | 24 |
| 16 | 15 |
| 17 | 18 |
| 18 | 22 |
| 19 | 40 |
| 20 | 40 |
| 21 | 17 |
| 22 | 18 |
| 23 | 6 |
| 24 | 20 |
| 25 | 24 |
| 26 | 30 |
| 27 | 5 |
| 28 | 6years |
| 29 | 25 |
| 30 | 25 |
| 31 | 15 |
| 32 | 4 |
| 33 | 7 |
| 34 | 15 |
| 35 | 15 |
| 36 | 9 |
| 37 | 5 |
| 38 | 10 |
| 39 | 11 |
| 40 | 0 |
| 41 | 8 |
| 42 | 27 |
| 43 | 13 |
| 44 | 10 |
| 45 | 35 |
| 46 | 15 |
| 47 | 25 |
| 48 | 5 |
| 49 | 25 |
| 50 | 2 |
| 51 | 37 |
| 52 | 5 |
| 53 | 10 |
| 54 | 14 |
| 55 | Over 20 |
| 56 | +20 |
| 57 | 5 |
| 58 | 20 |
| 59 | 22 years |
| 60 | 4 |
| 61 | 4 |
| 62 | 5 |
| 63 | 27 |
| 64 | 25 |
| 65 | 5 |
| 66 | 8 |
| 67 | 16 |
| 68 | 20 |
| 69 | 15 |
| 70 | 40 |
| 71 | 6 |
| 72 | 30 |
| 73 | 20 |
| 74 | 40 |
| 75 | 35 |
| 76 | 24 |
| 77 | 2 |
| 78 | 25 |
| 79 | 3 |
| 80 | 20 |
| 81 | 6 |
| 82 | 27 |
| 83 | 10 |
| 84 | 20 |
| 85 | 7 |
| 86 | 50y |
| 87 | 42 |
| 88 | 30 |
| 89 | 10 |
| 90 | 7 years |
| 91 | 7 |
| 92 | 10 |
| 93 | 0 |
| 94 | 2 |
| 95 | 10 |
| 96 | +20 |
| 97 | 20 |
| 98 | 12 |
| 99 | 5 |
| 100 | 7 |
| 101 | 20 |
| 102 | 10 |
| 103 | 5 |
| 104 | 20 |
| 105 | 25 |
| 106 | 4 |

| 1. **How much of your activity does knee replacement surgery represent?** | | |
| --- | --- | --- |
| Answer Choices | Responses | |
| < 10% | 6,60% | 7 |
| 11-30% | 25,47% | 27 |
| 31-50% | 25,47% | 27 |
| 51-70% | 26,42% | 28 |
| 71-90% | 8,49% | 9 |
| > 91% | 7,55% | 8 |
|  | **Answered** | **106** |
|  | **Skipped** | **24** |

| 1. **How many partial knee replacements (UKA, PFA, BiUKA, BCA) do you perform each year?** | | |
| --- | --- | --- |
| Answer Choices | Responses | |
| 0 | 4,67% | 5 |
| <10 | 21,50% | 23 |
| 11-30 | 32,71% | 35 |
| 31-50 | 22,43% | 24 |
| 51-100 | 11,21% | 12 |
| > 100 | 7,48% | 8 |
|  | **Answered** | **107** |
|  | **Skipped** | **23** |
| \| 1. **What is your ratio of partial knee replacements compared to all your knee replacements?** \| \| \| \| --- \| --- \| --- \| \| Answer Choices \| Responses \| \| \| 0% \| 4,67% \| 5 \| \| 1-10% \| 28,04% \| 30 \| \| 11-20% \| 25,23% \| 27 \| \| 21-30% \| 15,89% \| 17 \| \| 31-40% \| 14,02% \| 15 \| \| 41-50% \| 4,67% \| 5 \| \| > 51% \| 7,48% \| 8 \| \|  \| **Answered** \| **107** \| \|  \| **Skipped** \| **23** \| |  |  |

| 1. **What is your ratio of medial UKA according to all your PARTIAL knee replacements?** | | | |  |  |
| --- | --- | --- | --- | --- | --- |
| Answer Choices | Responses | | |  |  |
| 100% | 20,56% | | 22 |  |  |
| 91-100% | 32,71% | | 35 |  |  |
| 81-90% | 20,56% | | 22 |  |  |
| 61-80% | 14,95% | | 16 |  |  |
| < 60% | 11,21% | | 12 |  |  |
|  | **Answered** | | **107** |  |  |
|  | **Skipped** | | **23** |  |  |
| 1. **Do you utilize fixed-bearing UKA or mobile-bearing UKA?** | | | | | |
| Answer Choices | | Responses | | | |
| Only fixed-bearing | | 55,66% | | | 59 |
| Mainly fixed-bearing | | 15,09% | | | 16 |
| 50% - 50% | | 6,60% | | | 7 |
| Mainly mobile-bearing | | 10,38% | | | 11 |
| Only mobile-bearing | | 12,26% | | | 13 |
|  | | **Answered** | | | **106** |
|  | | **Skipped** | | | **24** |

| 1. **Why?** |  |  |
| --- | --- | --- |
| Answer Choices | Responses | |
| Better clinical outcome | 27,36% | 29 |
| I was trained on it | 34,91% | 37 |
| Lower failure rate | 12,26% | 13 |
| Higher risk of dislocation | 4,72% | 5 |
| Better kinematics | 9,43% | 10 |
| Other (please specify) | 11,32% | 12 |
|  | **Answered** | **106** |
|  | **Skipped** | **24** |

| 1. **Is your surgical time for a UKA shorter or longer compared to the one for a TKA?** | | |
| --- | --- | --- |
| Answer Choices | Responses | |
| Much shorter (> 30 minutes less) | 16,04% | 17 |
| Shorter (5 - 30 minutes less) | 65,09% | 69 |
| Same | 13,21% | 14 |
| Longer (5 - 30 minutes difference) | 2,83% | 3 |
| Much longer (> 30 minutes difference) | 2,83% | 3 |
|  | **Answered** | **106** |
|  | **Skipped** | **24** |

| 1. **How long does your patient stay in the hospital after unicompartmental knee replacement surgery?** | | |
| --- | --- | --- |
| Answer Choices | Responses | |
| No nights - Outpatient | 5,66% | 6 |
| One night | 30,19% | 32 |
| Two nights | 30,19% | 32 |
| Three nights | 22,64% | 24 |
| Four to six nights | 9,43% | 10 |
| More than six nights | 1,89% | 2 |
|  | **Answered** | **106** |
|  | **Skipped** | **24** |

| 1. **Do you use the same rehabilitation protocol for a UKA as for a TKA?** | | |
| --- | --- | --- |
| Answer Choices | Responses | |
| Yes | 44,34% | 47 |
| No - Shorter rehabilitation for UKA | 54,72% | 58 |
| No - Longer rehabilitation for UKA | 0,94% | 1 |
|  | **Answered** | **106** |
|  | **Skipped** | **24** |

| 1. **What do you include in your perioperative pain management protocol (more than one answer is possible)** | | |
| --- | --- | --- |
| Answer Choices | Responses | |
| Pre-op oral medicine | 39,62% | 42 |
| Pre-op regional blocks | 43,40% | 46 |
| Periarticular injections or local infiltration analgesia | 74,53% | 79 |
| Continuous post-op regional block | 19,81% | 21 |
|  | **Answered** | **106** |
|  | **Skipped** | **24** |

| 1. **Are you interested in custom-made UKA?** |  |  |
| --- | --- | --- |
| Answer Choices | Responses | |
| Yes, I use it | 4,72% | 5 |
| Yes, I could use it | 56,60% | 60 |
| No, I don't believe it would be useful | 25,47% | 27 |
| I have tried it, but I did not believe it would help me | 4,72% | 5 |
| Not at all | 8,49% | 9 |
|  | **Answered** | **106** |
|  | **Skipped** | **24** |

| 1. **Are you interested in robotics applied to UKA?** |  |  |
| --- | --- | --- |
| Answer Choices | Responses | |
| Yes, I use it | 14,95% | 16 |
| Yes, I could use it | 57,01% | 61 |
| No, I do not believe it would be useful | 17,76% | 19 |
| I have tried it, but I did not believe it would help me | 3,74% | 4 |
| Not at all | 6,54% | 7 |
|  | **Answered** | **107** |
|  | **Skipped** | **23** |

| 1. **What is your minimum varus alignment for medial UKA?** | |  |
| --- | --- | --- |
| Answer Choices | Responses | |
| I do not perform whole leg standing x-ray before UKA | 8,49% | 9 |
| I don´t care about preoperative alignment | 10,38% | 11 |
| Minimum 0°, no valgus | 35,85% | 38 |
| Minimum 3° varus | 31,13% | 33 |
| Minimum 5° varus | 14,15% | 15 |
|  | **Answered** | **106** |
|  | **Skipped** | **24** |

| 1. **Which is your varus deformity cut-off for considering a medial UKA?** | | |
| --- | --- | --- |
| Answer Choices | Responses | |
| 5° | 18,10% | 19 |
| 10° | 27,62% | 29 |
| 15° reducible | 30,48% | 32 |
| 15° | 7,62% | 8 |
| No cut off | 16,19% | 17 |
|  | **Answered** | **105** |
|  | **Skipped** | **25** |

| 1. **Which is your valgus deformity cut-off for considering a lateral UKA?** | | |
| --- | --- | --- |
| Answer Choices | Responses | |
| 5° | 26,21% | 27 |
| 10° | 32,04% | 33 |
| 15° reducible | 17,48% | 18 |
| 15° | 7,77% | 8 |
| No cut off | 16,50% | 17 |
|  | **Answered** | **103** |
|  | **Skipped** | **27** |
| \| 1. **Do you have a minimum age cut-off for considering a small implant in end stage compartimental OA?** \| \| \| \| --- \| --- \| --- \| \| Answer Choices \| Responses \| \| \| No \| 54,81% \| 57 \| \| No cut-off, only for patellofemoral replacements \| 7,69% \| 8 \| \| Yes, over 30 years old \| 2,88% \| 3 \| \| Yes, over 40 years old \| 13,46% \| 14 \| \| Yes, over 50 years old \| 18,27% \| 19 \| \| Yes, over 60 years old \| 2,88% \| 3 \| \|  \| **Answered** \| **104** \| \|  \| **Skipped** \| **26** \| |  |  |

| 1. **Do you have a hard cut-off on preoperative Body Mass Index (BMI) for implanting a small implant?** | | |
| --- | --- | --- |
| Answer Choices | Responses | |
| No | 47,12% | 49 |
| Yes, no small implants if BMI > 35 kg/m^2 | 29,81% | 31 |
| Yes, no small implants if BMI > 30 kg/m^2 | 19,23% | 20 |
| Yes, no small implants if BMI > 25 kg/m^2 | 3,85% | 4 |
|  | **Answered** | **104** |
|  | **Skipped** | **26** |

| 1. **Do you believe it is feasible to implant a UKA in an ACL-deficient knee?** | | |
| --- | --- | --- |
| Answer Choices | Responses | |
| No, never | 17,31% | 18 |
| Yes, always | 3,85% | 4 |
| Only in absence of subjective instability | 22,12% | 23 |
| Only in  older patients with primary antero(medial) OA and secondary degenerative ACL deficiency | 36,54% | 38 |
| Only if associated with an ACL reconstruction | 20,19% | 21 |
|  | **Answered** | **104** |
|  | **Skipped** | **26** |

| 1. **Do you believe it is feasible to implant an isolated UKA in a knee with a concomitant high-grade patellofemoral OA?** | | |  |  |  |
| --- | --- | --- | --- | --- | --- |
| Answer Choices | Responses | |  |  |  |
| No, better a TKA | 57,14% | 60 |  |  |  |
| No, better a UKA+PFA | 17,14% | 18 |  |  |  |
| Yes. no matter about patellofemoral OA | 19,05% | 20 |  |  |  |
| Yes, if a mobile-bearing UKA is utilized | 2,86% | 3 |  |  |  |
| Yes, only in male | 3,81% | 4 |  |  |  |
| Yes, only in female | 0,00% | 0 |  |  |  |
|  | **Answered** | **105** |  |  |  |
|  | **Skipped** | **25** |  |  |  |
| 1. **Which is your desired coronal alignment after a medial UKA (evaluated with long-standing X-rays)?** | | | | | |
| Answer Choices | | | | Responses | |
| The same as preoperative | | | | 3,81% | 4 |
| The same as pre-degeneration (therefore preserving constitutional varus) | | | | 51,43% | 54 |
| Neutral (0°) | | | | 15,24% | 16 |
| 1-5° varus | | | | 27,62% | 29 |
| 5-10° varus | | | | 0,95% | 1 |
| 1-5° valgus | | | | 0,95% | 1 |
|  | | | | **Answered** | **105** |
|  | | | | **Skipped** | **25** |

| 1. **Which is your desired coronal alignment after a lateral UKA (evaluated with long-standing X-rays)?** | | |
| --- | --- | --- |
| Answer Choices | Responses | |
| The same as preoperative | 5,71% | 6 |
| The same as pre-degeneration (therefore preserving constitutional valgus) | 48,57% | 51 |
| Neutral (0°) | 22,86% | 24 |
| 1-5° valgus | 21,90% | 23 |
| 5-10° valgus | 0,95% | 1 |
| 1-5° varus | 0,00% | 0 |
|  | **Answered** | **105** |
|  | **Skipped** | **25** |
